# The dosimetric effect of electron density overrides in 3DCRT Lung SBRT for a range of lung tumor dimensions

**DOI:** 10.1002/acm2.12446

**Published:** 2018-09-10

**Authors:** Grace E. A. Healy, Steven H. Marsh, Andrew T. Cousins

**Affiliations:** ^1^ University of Canterbury Canterbury New Zealand; ^2^ Department of Medical Physics and Bioengineering Christchurch Hospital Canterbury New Zealand

**Keywords:** density overrides, NSCLC, plan verification, SBRT, TPS

## Abstract

The combined effects of lung tumor motion and limitations of treatment planning system dose calculations in lung regions increases uncertainty in dose delivered to the tumor and surrounding normal tissues in lung stereotactic body radiotherapy (SBRT). This study investigated the effect on plan quality and accuracy when overriding treatment volume electron density values. The QUASAR phantom with modified cork cylindrical inserts, each containing a simulated spherical tumor of 15‐mm, 22‐mm, or 30‐mm diameter, was used to simulate lung tumor motion. Using Monaco 5.1 treatment planning software, two standard plans (50% central phase (50%) and average intensity projection (AIP)) were compared to eight electron density overridden plans that focused on different target volumes (internal target volume (ITV), planning target volume (PTV), and a hybrid plan (HPTV)). The target volumes were set to a variety of electron densities between lung and water equivalence. Minimal differences were seen in the 30‐mm tumor in terms of target coverage, plan conformity, and improved dosimetric accuracy. For the smaller tumors, a PTV override showed improved target coverage as well as better plan conformity compared to the baseline plans. The ITV plans showed the highest gamma pass rate agreement between treatment planning system (TPS) and measured dose (*P *<* *0.040). However, the low electron density PTV and HPTV plans also showed improved gamma pass rates (*P *<* *0.035, *P *<* *0.011). Low‐density PTV overrides improved the plan quality and accuracy for tumor diameters less than 22 mm only. Although an ITV override generated the most significant increase in accuracy, the low‐density PTV plans had the additional benefit of plan quality improvement. Although this study and others agreed that density overrides improve the treatment of SBRT, the optimal density override and the conditions under which it should be applied were found to be department specific, due to variations in commissioning and calculation methods.

## 1 INTRODUCTION

Lung Cancer is one of the world's most commonly diagnosed cancer types, as well as the most common cause of cancer death with an estimated 1.6 million deaths worldwide per year.^1^ Non‐small cell lung cancer (NSCLC) contributes to approximately 85% of all lung cancers.^2^

For patients whom surgery is not an option, conventional or stereotactic radiotherapy is frequently used.^3^, ^4^ One of the main toxicities stemming from radiation therapy in NSCLC is Radiation Pneumonitis (RP).^5^, ^6^ The use of stereotactic body radiotherapy (SBRT) to reduce the Planned Target Volume (PTV) margin and increase PTV edge dose gradients can improve local control and reduce the chance of toxicities such as RP.^7^

One of the issues associated with treating lung cancer with radiotherapy is motion of the tumor caused by patient breathing. In SBRT, this issue becomes an even greater challenge due to the addition of the smaller expansion of the PTV around the Internal Target Volume (ITV), with a steep dose gradient beyond this target volume.^8^ The most common technique for managing temporal tumor variation is four‐dimensional imaging, including respiration‐correlated 4‐Dimensional CT (4DCT) scanning,^9^ however, this can result in motion artifacts.^10^ Artifacts can be caused by irregular breathing traces, for example, coughing or patient motion during the scanning process.^11^

The presence of inhomogeneous media can also affect dose calculation accuracy. Several studies have examined the impact of different dose calculation algorithms on dose delivered to inhomogeneous media, in particular lung.^12^, ^13^, ^14^, ^15^ It is recommended that collapsed cone convolution (CCC) algorithms be used when complex algorithms such as Monte Carlo or Acuros XB (Varian Medical Systems, Palo Alto, CA) are not readily available.^12^, ^13^ One issue with CCC algorithms is that the model is unable to accurately calculate dose at the interface between lung and tumor.^12^ This is due to the assumption of transient charged particle equilibrium (TCPE) occurring at the tumor–lung interface not being true, and CCC algorithms cannot accurately model this effect.^15^ These errors have been shown to increase with smaller treatment volumes, where the ratio of tumor–lung interface surface area to tumor volume increases with decreasing target volume.^12^

During treatment, as the GTV moves through the ITV as defined by the 4DCT scan, the dose to the tumor will change compared to the treatment plan. As there is preferential dose buildup in higher density areas, as the GTV moves to a region of the ITV that is underdosed on the treatment plan, the GTV will receive a larger dose than expected.^14^, ^16^

One method to overcome the issues associated with inhomogeneity corrections in lung and tumor motion is to override the electron densities of the ITV/PTV.^17^, ^18^, ^19^ A study by Fu et al.^17^ devised a method for overriding the density of the PTV to 0.8 g/cm^3^ to reduce the planned MU while still delivering sufficient dose to the tumor for SBRT lung planning. Wiant et al.^18^ compared the use of free‐breathing CT scans, time average scans, and ITV/PTV tissue‐density overridden scans for lung SBRT to evaluate the accuracy of each method for predicting dose deposition in lung tissue. Accuracy was assessed from measurements using Gafchromic film in a QUASAR phantom.^18^ A further study by Wiant et al.^19^ looked at volumetric modulated arc therapy (VMAT) plans, and introduced a hybrid override with the ITV set to tumor density and PTV‐ITV set to an intermediate density.

The studies by Wiant et al.^18^, ^19^ were performed using the Eclipse planning system, and the method has not currently been extended to any other dose calculating algorithms. Also any implications due to tumors size or overriding the density of the PTV to a variety of low densities between lung and water have not been investigated. This study will look into quantifying the effect of density overrides to establish a trend based upon the relative sizes of the ITV and PTV.

## 2 METHODS

### 2.A Phantom study

To assess the impact of various density overrides on SBRT lung plans, a phantom study was conducted using the Elekta Synergy Linac and Monaco 5.1 TPS. A QUASAR Programmable Respiratory Motion Phantom (Modus Medical Devices Inc., London, ON, Canada) with modified inserts was used to simulate the craniocaudal motion of a lung tumor inside a SBRT patient.

**Figure 1:**
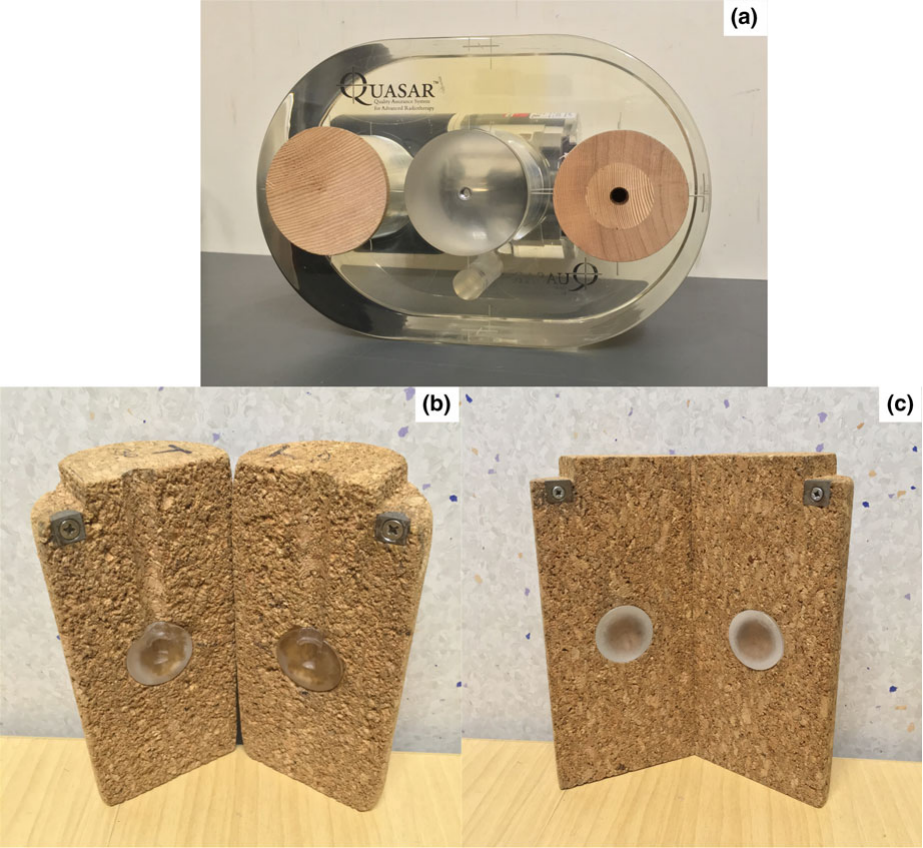
Above: The QUASAR^TM^ Programmable Respiratory Motion Phantom used at Christchurch Hospital, with a cedar ion chamber insert on the left and a cedar filler insert on the right. Below: The Cork‐based QUASAR‐compatible inserts. Left: 30‐mm CC13 insert. Right: 30‐mm film insert.

A study by Chang et al.^20^ compared the use of several lung substitute materials to reference lung material as listed in ICRU‐44. From this study, composition cork was found to be an acceptable lung substitute based on physical and dosimetric properties. QUASAR‐compatible inserts were designed and manufactured in‐house (Fig. 1). These included spherical PMMA tumor lesions with a density of 1.18 g/cm^−3^ and diameters of 15 mm, 22 mm, and 30 mm, corresponding to a range applicable to the lung tumor sizes treated clinically. Two types of inserts were designed, one for point dose measurements with a CC13 chamber and one for Gafchromic film.

A Siemens SOMATOM Definition CT scanner (Siemens Healthcare Limited) was used to acquire the 4DCT scans with the QUASAR phantom set to a 4.0‐s breathing period and 15‐mm motion amplitude. The respiratory waveform was simultaneously recorded with the Varian Real‐time Position Management (RPM) system. For each of the different cork inserts, the reconstruction process generated the average intensity projection (AIP) and a dataset containing each of the 10 respiratory phases from which the maximum intensity projection (MIP) was generated. The three required image datasets (AIP, MIP, and a 50% central phase dataset) were exported to the TPS.

The contouring of the two target volumes, the ITV and the PTV, was completed using the Monaco 5.1 TPS (Elekta, Inc) and utilized the MIP of each of the 4D CT scans for the different cork inserts. The PTV expansion was 1 cm in the superior/inferior direction and 0.5 cm in the axial plane. The contours were applied to each of the AIP and 50% central phase datasets for each insert.

A 3DCRT SBRT plan was developed on the 50% central phase datasets, with a prescription of 48 Gy in four fractions. Beam weightings were manually optimized using the van't Reit formula for Conformity Index.^21^

Three different electron density (ED) values were investigated corresponding to different proportions of lung and water. An average lung ED of 0.300 was used to determine the ED values for the different proportions. This value was consistent to the values seen clinically in our department, rounded to one significant figure. An average water ED of 1.000 was used. The overrides corresponded to 75% lung material and 25% water (ED = 0.475), 50% lung material and 50% water (ED = 0.650), and 25% lung material and 75% water (ED = 0.825). These were applied to the PTV, with or without the ITV set to an ED of 1.000 to form a hybrid plan. In total, eight override datasets were generated for each plan, and compared to the AIP and 50% central phase plans (Table 1). No beam weighting changes were applied with each override. The dose was rescaled to cover the PTV volume with the prescription dose set to the relative isoline of 80%.

**Table 1:** The Monaco 5.1 treatment plans investigated for each tumor size, and the corresponding relative electron density overrides applied

Plan type	ITV ED	PTV ED
50% Phase	n/a	n/a
AIP	n/a	n/a
ITV	1.000	n/a
PTV(0.475)	n/a	0.475
PTV(0.650)	n/a	0.650
PTV(0.825)	n/a	0.825
PTV(1.000)	n/a	1.000
HPTV(0.475)	1.000	0.475
HPTV(0.650)	1.000	0.650
HPTV(0.825)	1.000	0.825

ED, Electron Density (relative to water); 50% Phase, central phase of tumor motion treatment plan; AIP, Average Intensity Projection treatment plan; HPTV, Hybrid PTV overridden plan.

### 2.B Plan quality and coverage

To assess the relative coverage of each override plan compared to the baseline plans (AIP and 50% central phase), five PTV coverage metrics were assessed. This including the Mean Dose (Gy), Maximum Dose (Gy), Minimum Dose (Gy), D90, and D95. As these plans were forward planned using the collapsed cone algorithm, the Maximum and Minimum Doses (Gy) refers to dose at a point.

For Conformity and Heterogeneity, three Conformity Indices (CI) were used: van't Reits CI, CI(100%), and CI(50%). One metric for Heterogeneity Index (HI) was used.

#### 2.B.1 Conformity and heterogeneity indices

Monaco 5.1 uses the van't Riet formula for CI, which defines three volumes: the volume of the target receiving the prescribed dose (TV1), the target volume (TV), and the total volume of the prescription isodose (VR1).

(1) $$\mathrm{CI}=\frac{TV1^{2}}{TV\times VR1}$$

For CI(100%) and CI(50%), the CI(X%)‐type metrics indicated the ratio of the percentage (x) isodose volume (V_X%_) to the target volume (TV). The target volume in this case was the PTV.

(2) $$CI(X\%)=\frac{V_{X\%}}{\mathrm{TV}}$$

The Heterogeneity Index (HI) was also directly calculated from the DVH statistics. To calculate HI, the High Dose Reference value (HDR) and the Minimum Dose Reference value (MDR) were used to calculate Dx%, the dose that covers x% of the tissue. The standard Monaco 5.1 values for HDR and MDR were also used, corresponding to 95% and 5%.

(3) $$HI=\frac{D_{HDR\%}}{D_{MDR\%}}$$

### 2.C Dose verification

Two verification methods were used to compare the accuracy of the treatment plans: Ion Chamber Point Dose Measurements and EBT3 Gafchromic Film Dosimetry.

#### 2.C.1 Ion chamber point dose measurements

The point dose measurements were made with a 0.13‐cc IBA Dosimetry compact air ionization chamber (CC13). To compare the average charge collected by the ion chamber in the center of the tumor volume in the cork insert to the expected mean dose calculated by the TPS, a correction needed to be applied to the statistics reported by the TPS. This is due to the fact that the chamber remains fixed inside the tumor insert, but it moves relative to the PTV.

This results in the total dose being measured as an average across the volume that the active part of the ion chamber covers. As the amplitude of motion and the dimensions of the ion chamber cavity are both known, the active volume of the chamber was contoured as a structure in Monaco and the mean dose in Gy compared to that measured by the ion chamber.

#### 2.C.2 EBT3 gafchromic film dosimetry

EBT3 film was used in conjunction with the SNC Patient Film Analysis software (V6.6, Sun Nuclear Corporation) and an Epson Expression 11000XL scanner. As the tumor lesion moves inside the PTV on the TPS a reference frame correction was applied to each of the TPS Dose Planes. However, for the film measurement the film was fixed relative to the motion of the cork insert, and therefore the frame of reference coincides with the position of the tumor. A time‐weighted average correction was applied to each dose value in the TPS dose plane. As the motion of the GTV was sinusoidal with a fixed amplitude and period, the dose values only needed to be corrected in the direction of motion.

## 3 RESULTS

The effectiveness of each density override was assessed in two ways, including comparing the plan quality in the treatment planning system and the measurable aspects of the plan with point dose and radiochromatic film measurements.

### 3.A Plan quality and coverage

Comparison of the baseline plans (AIP and 50% central phase) to the density overridden plans for the 15‐mm insert shows that in every case the target coverage was at worst unchanged and mostly improved (Table 2). Aside from the ITV plan, the D95 value increased by 1.2 ± 0.4 Gy when compared to the 50% central phase plan. Consistent with the target coverage metrics, the ITV plan showed the least amount of difference between it and the 50% central phase and AIP plans in terms of the CI and HI.

**Table 2:** The target coverage DVH metrics and PTV conformity and heterogeneity metrics for a range of standard and electron density overridden treatment plans in a 15‐mm tumor object in a lung phantom

Plan type	DVH metrics (Gy)					Total MU delivered	CI			HI
	Mean	Max	Min	D90	D95		50%	100%	van't Reits	
50% Phase	12.7	15.0	9.4	11.6	11.2	1469.9	4.87	0.87	0.76	1.27
AIP	13.5	15.4	10.0	12.3	11.9	1548.5	5.36	1.16	0.78	1.26
ITV = 1.000	13.1	15.0	9.4	11.6	11.3	1460.2	4.82	0.89	0.79	1.31
PTV = 0.475	14.1	15.2	10.8	13.1	12.7	1449.6	4.79	1.18	0.86	1.18
PTV = 0.650	13.9	15.1	10.4	12.7	12.4	1494.5	5.08	1.18	0.83	1.20
PTV = 0.825	13.9	15.1	10.4	12.8	12.4	1457.8	4.87	1.15	0.85	1.20
PTV = 1.000	14.1	15.1	10.6	13.0	12.6	1447.7	4.80	1.15	0.86	1.19
HPTV = 0.475	13.6	15.0	10.1	12.4	12.0	1453.4	4.82	1.07	0.86	1.24
HPTV = 0.650	13.9	15.1	10.4	12.8	12.4	1450.7	4.82	1.13	0.86	1.21
HPTV = 0.825	14.4	15.1	10.6	13.0	12.6	1450.0	4.80	1.16	0.86	1.19

Plan Type format, ITV = 1.000 indicates the ITV is set to a relative electron density of 1.000.

CI, Conformity Index (50% isodose, 100% isodose, van't Reits formula); HI, Heterogeneity Index; 50% Phase, central phase of tumor motion treatment plan; AIP, Average Intensity Projection treatment plan; HPTV, Hybrid PTV overridden plan.

For the 22‐mm insert, less variation can be seen between the different density overrides compared to the 15‐mm insert (Table 3). For the PTV and HPTV plans, the D95 value increased by 1.1 ± 0.4 Gy when compared to the 50% central phase plan. Higher density overrides, specifically the HPTV = 0.475 plan, displayed the best CI and HI results.

**Table 3:** The target coverage DVH metrics and PTV conformity and heterogeneity metrics for a range of standard and electron density overridden treatment plans in a 22‐mm tumor object in a lung phantom

Plan Type	DVH metrics (Gy)					Total MU delivered	CI			HI
	Mean	Max	Min	D90	D95		50%	100%	van't Reits	
50% Phase	13.2	15.1	9.6	12.0	11.5	1435.3	4.37	1.00	0.80	1.28
AIP	13.5	15.2	9.7	12.2	11.7	1454.2	4.47	1.08	0.80	1.28
ITV = 1.000	13.5	15.3	9.6	12.0	11.6	1436.1	4.37	1.02	0.81	1.30
PTV = 0.475	14.0	15.1	10.2	12.9	12.4	1450.4	4.47	1.18	0.82	1.21
PTV = 0.650	14.1	15.2	10.3	13.0	12.6	1429.2	4.36	1.17	0.84	1.20
PTV = 0.825	14.3	15.4	10.4	13.2	12.8	1430.8	4.35	1.18	0.84	1.19
PTV = 1.000	14.4	15.5	10.7	13.3	12.9	1440.8	4.38	1.21	0.82	1.18
HPTV = 0.475	14.0	15.3	10.2	12.7	12.3	1436.4	4.38	1.14	0.84	1.23
HPTV = 0.650	14.2	15.4	10.4	13.0	12.6	1437.4	4.38	1.18	0.84	1.21
HPTV = 0.825	14.3	15.4	10.6	13.2	12.8	1439.1	4.38	1.20	0.83	1.19

Plan Type format, ITV = 1.000 indicates the ITV is set to a relative electron density of 1.000.

CI, Conformity Index (50% isodose, 100% isodose, van't Reits formula); HI, Heterogeneity Index; 50% Phase, central phase of tumor motion treatment plan; AIP, Average Intensity Projection treatment plan; HPTV, Hybrid PTV overridden plan.

For the 30‐mm insert, the average PTV and HPTV plans D95 value increased by 0.8 ± 0.3 Gy when compared to the 50% central phase plan (Table 4). The mean, minimum, and maximum doses showed less of an improvement as the insert size increased from 15 mm to 30 mm. No statistically significant difference was seen between CI (van't Riet) results (*P* < 0.06). The impact of density overrides is limited for large tumor volumes.

**Table 4:** The target coverage DVH metrics and PTV conformity and heterogeneity metrics for a range of standard and electron density overridden treatment plans in a 30‐mm tumor object in a lung phantom

Plan Type	DVH metrics (Gy)					Total MU delivered	CI			HI
	Mean	Max	Min	D90	D95		50%	100%	van't Reits	
50% Phase	13.8	15.3	9.6	12.6	12.3	1414.1	4.64	1.22	0.80	1.22
AIP	13.8	15.4	9.5	12.6	12.2	1399.9	4.53	1.19	0.81	1.24
ITV = 1.000	13.9	15.5	9.6	12.6	12.2	1403.6	4.57	1.20	0.81	1.24
PTV = 0.475	14.5	15.7	10.2	13.6	13.3	1410.3	4.56	1.31	0.78	1.16
PTV = 0.650	14.2	15.3	9.9	13.2	12.9	1406.1	4.62	1.28	0.78	1.17
PTV = 0.825	14.3	15.4	9.9	13.4	13.0	1392.2	4.53	1.27	0.79	1.17
PTV = 1.000	14.4	15.6	10.1	13.5	13.2	1398.0	4.53	1.28	0.79	1.16
HPTV = 0.475	14.2	15.5	9.9	13.1	12.8	1405.0	4.57	1.26	0.79	1.20
HPTV = 0.650	14.4	15.6	10.0	13.4	13.1	1406.5	4.57	1.29	0.78	1.17
HPTV = 0.825	14.5	15.7	10.2	13.6	13.2	1408.4	4.56	1.30	0.78	1.16

Plan Type format, ITV = 1.000 indicates the ITV is set to a relative electron density of 1.000.

CI, Conformity Index (50% isodose, 100% isodose, van't Reits formula); HI, Heterogeneity Index; 50% Phase, central phase of tumor motion treatment plan; AIP, Average Intensity Projection treatment plan; HPTV, Hybrid PTV overridden plan.

### 3.B Plan verification

#### 3.B.1 Point dose measurements

The majority of the point dose measurements were within the clinically accepted tolerance of ±3% compared to the dose calculated by the TPS. For each result, a Student's t test was performed to compare the statistical significance of the ED override results as compared to the baseline plan (50% central phase).

**Figure 2:**
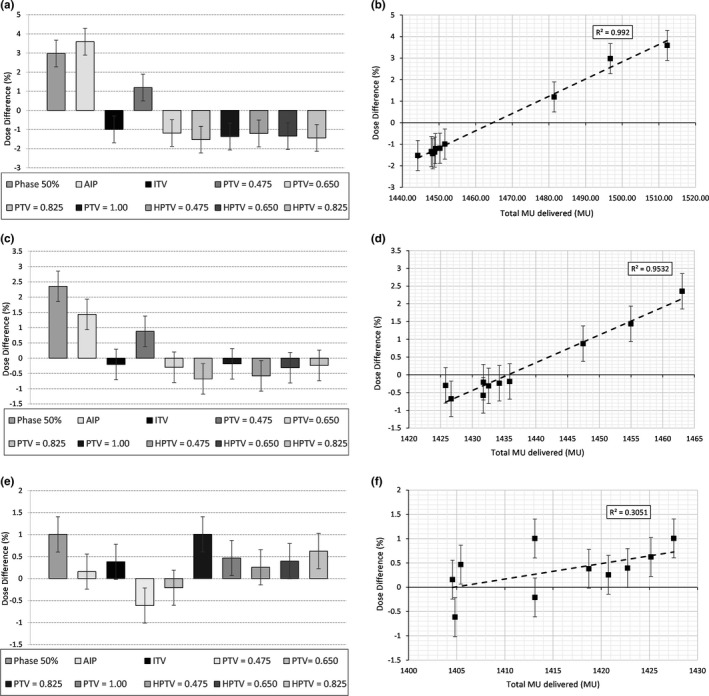
The difference between the average doses to the ion chamber structure reported by Monaco and the measured dose to the ion chamber, for each of the baseline and density overridden plans for the cork inserts. (a) Percentage Difference, 15‐mm insert, (b) MU difference, 15‐mm insert, (c) Percentage Difference, 22‐mm insert, (d) MU difference, 22‐mm insert, (e) Percentage Difference, 30‐mm insert, and (f) MU difference, 30‐mm insert.

For the 15‐mm insert, all ED overridden plans showed a significant (*P* < 0.05) difference to the 50% central phase plan (Fig. 2). The largest percentage dose differences were for the 50% central phase and AIP plans. A positive trend was displayed between percentage difference and total MU (R^2^ = 0.992).

For the 22‐mm insert, the best‐performing ED overrides were the ITV = 1.00 plan and PTV = 1.00 plan (*P* < 0.003, *P* < 0.002). The positive trend that was previously seen in the 15‐mm result remained between the percentage difference and total MU (R^2^ = 0.9532), however, the correlation was weaker.

For the 30‐mm insert, there was no density overridden plan that showed a statistically significant improvement over the original plans (average *P* < 0.232). The positive trend seen between the percentage difference and total MU was not significant (R^2^ = 0.3051). As there was limited benefit seen in the TPS and point dose results, film work was not completed for the 30‐mm tumor insert.

#### 3.B.2 Gafchromic film

Three absolute dose gamma analysis results were obtained (Criteria of 1%/1 mm, 2%/2 mm, and 3%/3 mm) across two dose threshold levels (10% and 40%). The plans that were most effective in the ion chamber measurements were repeated to decrease the amount of film required. The HPTV = 0.650 plan was used as there was minimal difference shown in the ion chamber measurements between HPTV plans. Forty percent was chosen to minimize the effect of the sudden dose difference at the edge of the film. For the 15‐mm insert, the 50% central phase plan was shown to have the lowest mean gamma pass rates across all criteria.

**Figure 3:**
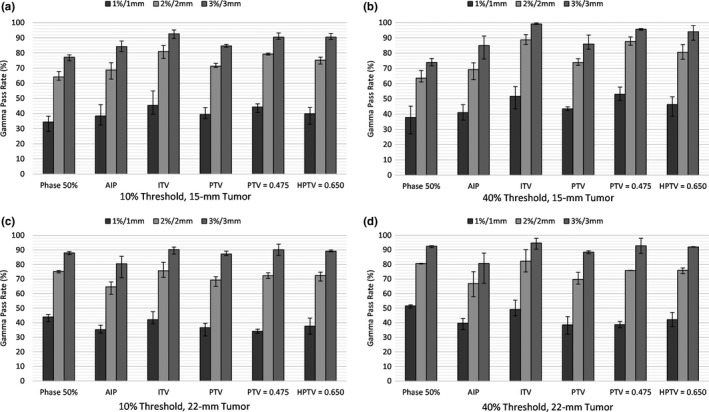
The mean gamma pass rates (%) and range (±%) between the Gafchromic film and TPS dose distributions for the (a) 10% Threshold, 15‐mm tumor insert, (b) 40% Threshold, 15‐mm tumor insert, (c) 10% Threshold, 22‐mm tumor insert, and (d) 40% Threshold, 22‐mm tumor insert.

The best‐performing plan was the ITV override, which showed a 3%, 3‐mm pass rate of 92.7% for the 10% threshold, and 99.7% for the 40% threshold (Fig. 3). For the gamma criterion 2%/2 mm and 3%/3 mm, the ITV, PTV = 0.475, and HPTV = 0.650 plans all showed significant differences to the baseline plans (*P* < 0.011).

For the 22‐mm insert, the best‐performing plan was the ITV override, which showed a 3%, 3‐mm pass rate of 90.2% for the 10% threshold, and 94.7% for the 40% threshold gamma criteria. However, for the 10% TH gamma criteria, no plans showed a consistent significant difference to the baseline plan.

## 4 DISCUSSION

The key motivation for exploring the effect of ED overrides was to improve the treatment of lung cancer with SBRT. This can be achieved in two different ways: improving the quality of the treatment plan, in terms of target coverage and conformity, and improving the accuracy of the treatment delivery.

Overriding the ED of a target volume to a water‐equivalent ED is associated with better target coverage due to the fact that the CCC algorithm accounts for inhomogenieties by scaling dose kernels by the relative electron densities.^22^, ^23^ Previous studies have shown that for the CCC algorithm, a lower lung ED is associated with a reduced medium target dose.^24^ This is consistent with results seen for the PTV and HPTV overrides. Across all inserts, the planning aim of 95% of the PTV volume receiving the prescribed dose of 12 Gy per fraction was achieved. In addition, none of these plans achieved unacceptably high maximum dose values.

The ideal CI value is 1.0, whereas an acceptable HI is anything <2.0 based on the Radiation Therapy Oncology Group (RTOG) guidelines for SBRT. For all plans, the HI was found to be less than 2. This was expected as there were no irregular shapes in this study or OARs to avoid. Therefore, delivering a homogeneous dose to the PTV was less of an issue. Varying levels of agreement were seen in the ten plans investigated for the 15‐mm, 22‐mm, and 30‐mm inserts. While the 15‐mm and 22‐mm results showed positive correlation between the prescribed MU and the dose measured by the ion chamber, the same cannot be said for the 30‐mm insert. While there was an improvement in dosimetric accuracy seen using plans other than the 50% central phase plan, all results were within 1% of the expected dose, well below the acceptable clinical limit of 2%. There is a limited application for applying density overrides to large tumors.

The planning system performs adequately for calculating dose to large tumors, where the effect of the uncertainty of heterogeneity accounting algorithms is limited. Little difference was seen for the 15‐mm and 22‐mm inserts for the higher density range of overrides. Less fluence would be required in higher densities to generate the same desired dose coverage, resulting in fewer MU being delivered. Although the ion chamber measurements are useful in terms of absolute dose, there is limited value to the ion chamber results as the dose measured is for only a small portion of the ITV. For both the 15‐mm and 22‐mm results, the ITV plan showed the high gamma pass rates for both the 2%/2 mm and 3%/3 mm criteria. Profiles through the center of the tumor confirm improved agreement between the ITV TPS dose plane and measured film dose plane.

When the density of the ITV or PTV is overridden closer to the ED of the GTV, less fluence is required to deliver the prescribed dose as conformal dose deposition is more achievable in higher ED volumes. The lower fluence results in a reduction in the total MU prescribed when all other beam parameters are kept constant.

When this plan is delivered, however, regardless of the position of the GTV inside the ITV, a higher proportion of the energy fluence will be deposited inside the higher electron density tumor as compared to the surrounding lung material. As the tumor moves within the ITV, the result is a higher dose overall to the ITV. As the baseline 50% central phase plan is unable to predict this smearing out effect, the measured dose in the center of the ITV will be higher than expected, a result that was consistently seen in both the ion chamber and film measurements. The impact of the dose smearing effect is not only seen in the ITV plan, but the lower density PTV and HPTV plans as well.

Previously published work most comparable to this study is the study by Wiant et al.^19^ In their study, a large diameter tumor object was used, and the CI and mean dose values were all approximately the same, with the only significant difference occurring with the maximum dose to the PTV. The ITV plan showed the greatest increase in maximum dose compared to the 50% central phase plan, whereas the PTV and hybrid plans showed minimal differences. This is not consistent with the plan quality results seen in this study. A reason for the variation between results may stem from the differences between the two planning systems and the operation of the heterogeneity accounting algorithms.

In both studies, density overrides were shown to improve dosimetric accuracy, but in this study density overrides were shown to be clinically beneficial for tumors less than 22 mm. An additional study into the mid‐sized range around 22 mm would be beneficial. Applying an established Lung SBRT planning protocol to a small selection of override options is necessary to determine the clinic‐specific best fit.

## 5 CONCLUSION

No significant statistical difference was seen between the 50% central phase and AIP plans. A trend was demonstrated where, for smaller tumors (<22‐mm diameter), the geometric and dosimetric dose coverage and conformity improved when a PTV override was applied. For larger tumors (>22‐mm diameter), minimal differences were seen in terms of plan quality and accuracy, suggesting the results are equipment specific. Improvements to dosimetric accuracy were seen as the tumor size decreased. The results established in this study suggest a valid method for improving outcomes to patients with NSCLC treated with SBRT, particularly for small tumors where dosimetric as well as geometric accuracy is a greater concern.

## CONFLICT OF INTEREST

The authors declare no conflicts of interest.
